# Combining Whole-Genome Sequencing and Multimodel Phenotyping To Identify Genetic Predictors of *Salmonella* Virulence

**DOI:** 10.1128/mSphere.00293-20

**Published:** 2020-06-10

**Authors:** Alanna Crouse, Catherine Schramm, Jean-Guillaume Emond-Rheault, Adrian Herod, Maud Kerhoas, John Rohde, Samantha Gruenheid, Irena Kukavica-Ibrulj, Brian Boyle, Celia M. T. Greenwood, Lawrence D. Goodridge, Rafael Garduno, Roger C. Levesque, Danielle Malo, France Daigle

**Affiliations:** a McGill Research Center on Complex Traits, McGill University, Montreal, Quebec, Canada; b Department of Human Genetics, McGill University, Montreal, Quebec, Canada; c Lady Davis Institute for Medical Research, Montreal, Quebec, Canada; d Institute for Integrative and Systems Biology, Faculty of Medicine, Université Laval, Quebec City, Quebec, Canada; e Department of Microbiology and Immunology, Dalhousie University, Halifax, Nova Scotia, Canada; f Département de Microbiologie, Infectiologie et Immunologie, Université de Montréal, Montreal, Quebec, Canada; g Department of Microbiology and Immunology, McGill University, Montreal, Quebec, Canada; h Food Science Department, University of Guelph, Guelph, Ontario, Canada; i Canadian Food Inspection Agency, Dartmouth Laboratory, Dartmouth, Nova Scotia, Canada; j Swine and Poultry Infectious Diseases Research Center (CRIPA-FRQNT), Montreal, Quebec, Canada; U.S. Food and Drug Administration

**Keywords:** *Salmonella*, amoeba, epithelial cells, food safety, host cell interaction, macrophages, prediction, systemic infection, virulence, whole-genome sequencing

## Abstract

*Salmonella* comprises more than 2,600 serovars. Very few environmental and uncommon serovars have been characterized for their potential role in virulence and human infections. A complementary *in vitro* and *in vivo* systematic high-throughput analysis of virulence was used to elucidate the association between genetic and phenotypic variations across *Salmonella* isolates. The goal was to develop a strategy for the classification of isolates as a benchmark and predict virulence levels of isolates. Thirty-five phylogenetically distant strains of unknown virulence were selected from the *Salmonella* Foodborne Syst-OMICS (SalFoS) collection, representing 34 different serovars isolated from various sources. Isolates were evaluated for virulence in 4 complementary models of infection to compare virulence traits with the genomics data, including interactions with human intestinal epithelial cells, human macrophages, and amoeba. *In vivo* testing was conducted using the mouse model of *Salmonella* systemic infection. Significant correlations were identified between the different models. We identified a collection of novel hypothetical and conserved proteins associated with isolates that generate a high burden. We also showed that blind prediction of virulence of 33 additional strains based on the pan-genome was high in the mouse model of systemic infection (82% agreement) and in the human epithelial cell model (74% agreement). These complementary approaches enabled us to define virulence potential in different isolates and present a novel strategy for risk assessment of specific strains and for better monitoring and source tracking during outbreaks.

**IMPORTANCE** *Salmonella* species are bacteria that are a major source of foodborne disease through contamination of a diversity of foods, including meat, eggs, fruits, nuts, and vegetables. More than 2,600 different Salmonella enterica serovars have been identified, and only a few of them are associated with illness in humans. Despite the fact that they are genetically closely related, there is enormous variation in the virulence of different isolates of Salmonella enterica. Identification of foodborne pathogens is a lengthy process based on microbiological, biochemical, and immunological methods. Here, we worked toward new ways of integrating whole-genome sequencing (WGS) approaches into food safety practices. We used WGS to build associations between virulence and genetic diversity within 83 *Salmonella* isolates representing 77 different *Salmonella* serovars. Our work demonstrates the potential of combining a genomics approach and virulence tests to improve the diagnostics and assess risk of human illness associated with specific *Salmonella* isolates.

## Abstract (precis)

*Salmonella* species are bacteria that are a major source of foodborne disease through contamination of a diversity of foods, including meat, eggs, fruits, nuts, and vegetables. More than 2,600 different Salmonella enterica serovars have been identified, and only a few of them are associated with illness in humans. Despite the fact that they are genetically closely related, there is enormous variation in the virulence of different isolates of Salmonella enterica. Identification of foodborne pathogens is a lengthy process based on microbiological, biochemical, and immunological methods. Here, we worked toward new ways of integrating whole-genome sequencing (WGS) approaches into food safety practices. We used WGS to build associations between virulence and genetic diversity within 83 *Salmonella* isolates representing 77 different *Salmonella* serovars. Our work demonstrates the potential of combining a genomics approach and virulence tests to improve the diagnostics and assess risk of human illness associated with specific *Salmonella* isolates.

## INTRODUCTION

Salmonella enterica is a facultative intracellular Gram-negative bacterium that remains one of the major sources of foodborne disease for human populations throughout the world (1). *Salmonella* contaminates a diversity of foods of animal origin (eggs, poultry, beef, and pork) but also fresh produce. Salmonella enterica comprises more than 2,600 different serovars classified into six subspecies, with subspecies *enterica* being responsible for 99% of *Salmonella* infections in humans (2, 3). These serovars differ in their adaptation to various hosts and the diseases they cause. Typhoidal serovars cause a systemic life-threatening disease (enteric fever) in humans. On the other hand, nontyphoidal *Salmonella* (NTS) serovars are capable of causing disease in a broad range of hosts (mammals, reptiles, and birds). In healthy humans, infection with NTS serovars normally develops into a localized self-limiting intestinal inflammation called salmonellosis (4, 5). When the host immunity is compromised, NTS can also cause fatal infections (6).

Although *Salmonella* imposes a major burden to public health, not all serovars are equally harmful and have the same levels of virulence. While a few serovars are inclined to cause severe extraintestinal systemic disease, others induce mild gastroenteritis and still others survive within the host without causing disease (7, 8). Despite the potential differences in risk to human health, all serovars isolated from food products are treated the same during food safety procedures. Benchmarking virulence combined with serotyping and its potential in foodborne disease diagnostics could lead to a reshaping of current food safety practices to recognize differences in isolate virulence and risk that could help decrease food waste while allowing public health officials to estimate the scope and impact of *Salmonella* outbreaks.

Given that genetic heterogeneity underlies phenotypic diversity, elucidating the genetic determinants and gene content involved in *Salmonella* virulence is an important step toward understanding the clinical relevance of particular serovars and their potential role in disease manifestation. It has been well established that *Salmonella* pathogenesis is a highly complex process which relies on a concerted effort from multiple virulence factors. More than 21 *Salmonella* pathogenicity islands (SPIs) containing various virulence-associated genes have been described. These functions, in addition to virulence-associated genes located outside SPIs and within virulence plasmids, can be gained and lost (9, 10). The high degree of genome, serovar, and virulence diversity within Salmonella enterica heightens the complexity of *Salmonella* infection, since different serovars contain different collections of SPIs in different locations (10, 11). Despite the extensive study into the genetics of *Salmonella* pathogenicity, many virulence-associated genes and their contributions to virulence remain unknown. Furthermore, much of the progress made to date has been based on a select group of *Salmonella* serovars, underrepresenting the diversity of strains associated with food products.

In humans, *Salmonella* enters the body following ingestion of contaminated food or water. It survives the harsh acidic conditions of the stomach and reaches the intestine where it adheres to the intestinal epithelial cells, causing a localized infection to the terminal ileum, colon, and mesenteric lymph nodes. In immunocompromised individuals, nontyphoidal serovars can cross the intestinal barrier, invade and replicate within macrophages, and then disseminate systemically (12). There are several approaches to model *Salmonella* virulence. Intestinal epithelial cells and macrophages have been widely used to test *Salmonella* virulence factors and study host-*Salmonella* interaction (13). The amoeba is a complementary *in vitro* single cell assay and a potentially useful model to study host-*Salmonella* interaction (14, 15). This is particularly relevant, since amoeba represent a key reservoir of *Salmonella* for survival in the environment and play a significant role in transmission to humans (16). The mouse is the most widely used animal model for *Salmonella* infection, and several models have been developed to study distinct aspects of human *Salmonella* infection *in vivo* (17).

In the present study, we report the use of a multimodel phenotyping approach and whole-genome sequencing (WGS) to assess 83 *Salmonella* isolates representing 77 serovars, providing a repertoire of phenotypically and genotypically defined NTS strains. Comparative genomics and 4 host models were used to determine how genomic variations between isolates may influence phenotypic traits implicated in virulence, pinpoint novel virulence markers, and predict virulence both *in vitro* and *in vivo*.

## RESULTS

### Identification of phylogenetically distant *Salmonella* isolates.

**FIG 1:**
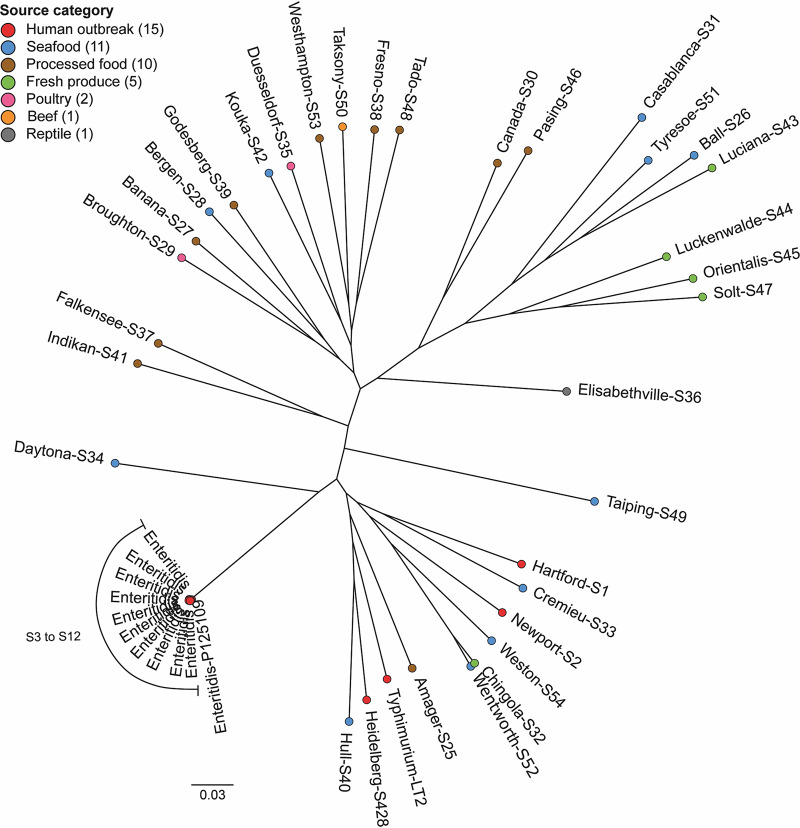
Molecular phylogeny of 35 Salmonella enterica serovar isolates. A phylogenetic tree was constructed based on the whole-genome sequences of 35 Salmonella enterica serovars. Serovar font color indicates strain source category. Number of SNPs between two isolates is represented by the distance between two colored dots. The 0.03 scale is used as a unit and indicates the number of nucleotide substitutions per site.

Salmonella enterica serovars have remarkable biological and genomic diversity. We first selected phylogenetically distant *Salmonella* serovars to evaluate their strain-specific virulence. Thirty-five isolates were selected from the SalFoS database (https://salfos.ibis.ulaval.ca), maximizing genetic diversity and range of sources, including fresh produce. The aim was to estimate whether it was possible to link genetic differences to phenotypic characteristics (Fig. 1, Table 1). In addition to these 35 isolates, 8 S. enterica serovar Enteritidis strains associated with human illness and 2 reference genomes from NCBI, S. enterica serovar Typhimurium LT2 (GenBank accession number NC_003197.2) and *S.* Enteritidis P125109 (GenBank accession number NC_011294.1), were included in the phylogenetic analysis. The pan-genome was evaluated using SaturnV (v1.1.0) to determine genes present in all genomes and to compute a core single nucleotide polymorphism (SNP) tree (18). The pan-genome is defined as all genes of a clade. For instance, this concept can be applied to species, genus, or even to a domain of life. The pan-genome is separated into 3 groups: core genes (present in all isolates), flexible genes (found in more than one isolate, but not in all of them), and unique genes (only found in one isolate) (19). The pan-genome of the 45 S. enterica genomes contains 3,100 core genes, 3,612 flexible genes, and 3,704 unique genes for a total of 10,461 genes. The phylogenetic tree was computed based on 168,292 core SNPs. All S. enterica serovars from human outbreaks, except for *S.* Enteritidis, share a more recent common ancestor and thus are more related. Of interest, 4 of the 5 S. enterica serovars isolated on fresh produce also share a recent common ancestor (*S.* Luciana, *S.* Luckenwalde, *S.* Orientalis, and *S.* Solt).

**TABLE 1:** 35 *Salmonella* strains of unknown virulence tested in 4 models of infection

Isolate ID	Serotype	Source category	Host organism	Specific source
S1	Hartford	Human	Human	
S2	Newport	Human	Human	
S3	Enteritidis	Human	Human	
S5	Enteritidis	Human	Human	
S25	Amager	Other		Animal feed
S26	Ball	Fish/shellfish	Shrimp	
S27	Banana	Other		Animal feed
S28	Bergen	Fish/shellfish	Shrimp	
S29	Broughton	Poultry		
S30	Canada	Other		Chocolate
S31	Casablanca	Fish/shellfish	Shrimp	
S32	Chingola	Plant (fruit/veg)	Seaweed	
S33	Cremieu	Fish/shellfish	Eel	
S34	Daytona	Fish/shellfish	Clams	
S35	Duesseldorf	Poultry		
S36	Elisabethville	Animal/other	Agamid	
S37	Falkensee	Other		Rice seasoning
S38	Fresno	Other		Animal feed
S39	Godesberg	Nuts/seeds	Sesame	Halawa
S40	Hull	Fish/shellfish	Conch	
S41	Idikan	Nuts/seeds	Sesame	Tahini
S42	Kouka	Fish/shellfish	Oysters	
S43	Luciana	Plant (fruit/veg)	Cantaloupe	
S44	Luckenwalde	Plant (fruit/veg)	Cocoa	Beans
S45	Orientalis	Nuts/seeds	Alfalfa	Seeds
S46	Pasing	Other		Chocolate
S47	Solt	Plant (fruit/veg)	Cocoa	Beans
S48	Tado	Other		Animal feed
S49	Taiping	Fish/shellfish	Fish	
S50	Taksony	Animal/other	Ox	
S51	Tyresoe	Fish/shellfish	Shrimp	
S52	Wentworth	Fish/shellfish	Cuttlefish	
S53	Westhampton	Other		Animal feed
S54	Weston	Fish/shellfish	Shrimp	
S428	Heidelberg	Human	Human	Feces

### Comparative virulence of 35 phylogenetically distant *Salmonella* isolates in four models of infection.

Phenotypic evaluation of isolate virulence was conducted using 4 models of infection: (i) human epithelial cells (bacterial adhesion, invasion, and survival), (ii) human macrophages (phagocytosis and intracellular survival at 2 and 18 h postinfection), (iii) amoeba (intracellular survival), and (iv) *in vivo* mouse models of systemic infection. In all cases, bacterial load was used as a marker of virulence. For each model, the reference strain *S.* Typhimurium SL1344 was used as a high-load control, and its isogenic mutant for the genes *invA* (SPI-1) and *sseB* (SPI-2) (here referred to as the Δ*invA* Δ*sseB* mutant) was used as a low-load control, as this strain exhibits impaired host cell entry and intracellular survival.

For *in vivo* phenotyping, C57BL/6J mice were chosen because they are highly susceptible to infection and develop clinical disease within 3 to 5 days postinoculation with virulent *Salmonella* Typhimurium (20). We chose not to use antibiotics, since it may interfere with *Salmonella* growth. We then tested two routes of inoculation, oral (p.o.) and intravenous (i.v.), with the aim of identifying a robust phenotype that would provide enough statistical power to allow phenotypic discrimination among the different *Salmonella* isolates tested. C57BL/6J mice were inoculated with 10^5^ (i.v.) or 10^7^ (p.o.) CFU, and bacterial loads were measured in spleen, liver, and feces (oral infection only) 3 days postinoculation. We observed a significant difference in tissue bacterial loads between SL1344- and Δ*invA* Δ*sseB* mutant-inoculated mice (see Fig. S1A and B in the supplemental material). The difference was greater and more significant when mice were inoculated i.v. (5-log difference for the spleen with i.v. injection compared to a 2-log difference with oral inoculation). There was no difference in fecal excretion (Fig. S1A). We then tested a subset of isolates with unknown virulence (Fig. S1C and D). The 4 isolates tested showed similar phenotypes when inoculated orally or i.v.; high bacterial loads were detected for isolates S3 and S5 and lower bacterial loads for S35 and S39. Since the i.v. inoculation route gave a more robust phenotype in terms of tissue load difference between the two control strains and a less variable phenotype, this was the route of infection that was selected for the following experiments.

10.1128/mSphere.00293-20.1 FIG S1 Comparison of oral and systemic Salmonella enterica infection in C57BL/6J mice. Mice were either inoculated per os (A and C) or intravenously (B and D). Bacterial loads in the spleens and livers of C57BL/6J mice at day 3 after oral (A and C) and systemic (B and D) *Salmonella* infection. The graphs represent the CFU counts per weight of organs or feces derived from the means ± standard errors of the means (SEMs) from one experiment. Data are representative of two independent experiments. Two-way ANOVA with Sidak’s multiple-comparison test was used to assess significance. **, *P* ≤ 0.01; ****, *P* < 0.0001. Download FIG S1, PDF file, 0.1 MB. © Crown copyright 2020. 2020 Crown This content is distributed under the terms of the Creative Commons Attribution 4.0 International license.

**FIG 2:**
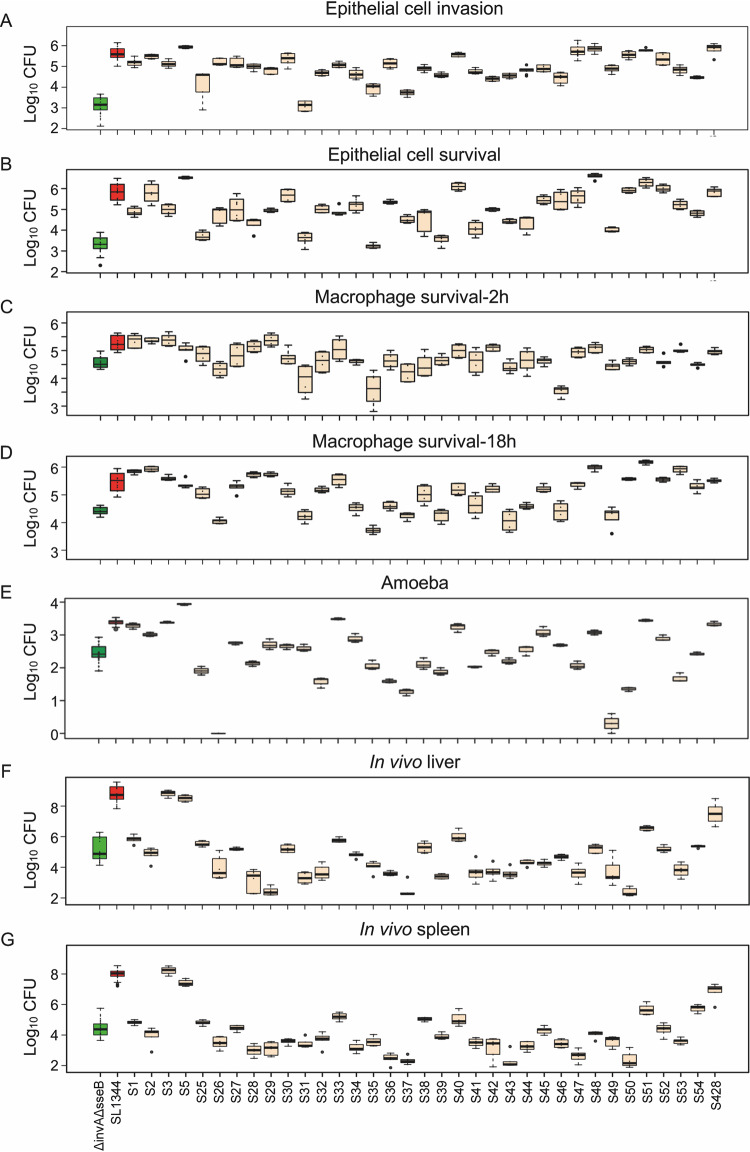
Phenotyping 35 phylogenetically distant *Salmonella* serovars in four models of infection. Bacterial load after infection was used as a marker of virulence. Infection of human epithelial cells (A and B), macrophages (C and D), amoebas (E), and *in vivo* mouse models of systemic disease (F and G). Human epithelial cells were used to measure *Salmonella* virulence in the form of adhesion, invasion, and replication within epithelial cells. Shown here are invasion (A) measured by intracellular bacterial burden (CFU) of cells 180 min postinfection and survival (B) as measured 18 h postinfection. Human macrophages were used to test strain propensity for phagocytosis and survival measured at 2 (C) and 18 h (D) postinfection. (E) Virulence in the amoeba model was measured by intracellular bacterial burden. *In vivo* virulence was measured by bacterial burden in mouse liver (F) and spleen (G) 3 days postinfection. Bacterial burden (CFU) in the spleen is shown here. Sample size varied across models and isolates. Either 2, 3, or 4 biological replicates with 3 technical replicates per isolate were used for epithelial cells. Either 2 or 3 biological replicates with 3 technical replicates were used for macrophages. Three biological replicates were performed per isolate in the amoeba and six biological replicates were performed per isolate in the mouse. In all cases, Log_10_ bacterial load was used as a marker of virulence. The distribution of data for each serovar is shown as a box plot. Low-virulence control Δ*invA* Δ*sseB* strain is shown in green, high-virulence control SL1344 is shown in red, and test strains are in beige.

**FIG 3:**
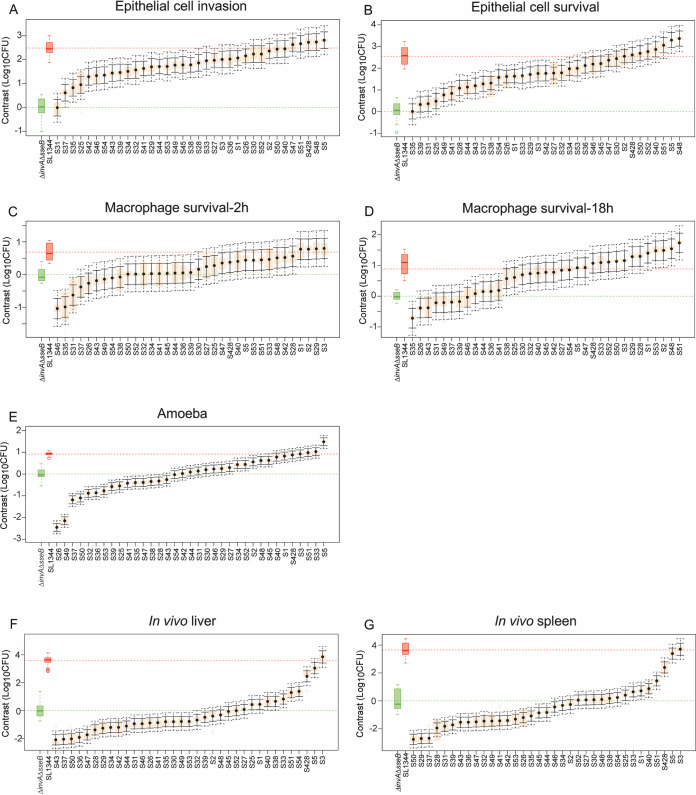
Distribution of contrasts in the four models of infection for all 35 phylogenetically distant *Salmonella* serovars. Contrast analysis was used to compare differences in bacterial burden caused by each of the 35 serovars against the low-virulence control Δ*invA* Δ*sseB* strain (mean value shown in green) within all phenotypic traits measured in each model. Epithelial cell invasion (A) and survival (B), macrophage phagocytosis (C) and survival (D), replication in amoeba (E), and bacterial load in liver (F) and spleen (G) of mice are shown. Isolate means are shown by black dots. Orange boxes denote the ranges of values, solid black bars show the 95% confidence intervals, and dotted black bars show the confidence intervals when adjusted for multiple testing using Bonferroni’s correction. All isolates whose adjusted confidence intervals do not cross the green line were significantly different from the Δ*invA* Δ*sseB* strain (*P* < 0.05). Either 2, 3, or 4 biological replicates with 3 technical replicates per isolate were used for epithelial cells. Either 2 or 3 biological replicates with 3 technical replicates were used for macrophages. Three biological replicates were performed per isolate in the amoeba and six biological replicates were performed per isolate in the mouse.

All models showed a wide distribution of phenotypes across the 35 isolates (Fig. 2). Of all phenotypic traits measured, epithelial cell adhesion and macrophage phagocytosis showed the least diversity, with most of the strains not differing significantly from the controls in terms of bacterial adhesion to epithelial cells or uptake by macrophages (see Fig. S2). Contrast analysis was used to compare the differences in bacterial burden caused by each of the 35 isolates against the low control Δ*invA* Δ*sseB* strain within all phenotypic traits measured in each model (Fig. 3). Regarding the epithelial cell model, the majority of the isolates showed mean values that were significantly higher than those for the low control Δ*invA* Δ*sseB* strain, for both invasion and survival (Fig. 3A and B). In macrophages, a wider distribution of CFU was observed, even if the difference between both controls was smaller. At 2 h postinfection, 3 isolates had lower bacterial load than the Δ*invA* Δ*sseB* control, 27 isolates showed no difference compared to the Δ*invA* Δ*sseB* strain, and 5 were higher that the Δ*invA* Δ*sseB* control (Fig. 3C). For the bacterial loads observed 18 h postinfection, 23 isolates were higher than the Δ*invA* Δ*sseB* isolate, 11 isolates were similar to the control, and 1 isolate was lower (Fig. 3D). The amoeba and mouse models showed the most phenotypic discrimination, including many isolates with lower bacterial burden than the Δ*invA* Δ*sseB* control. In the amoeba model, 14 isolates caused bacterial burdens lower than the Δ*invA* Δ*sseB* strain, 8 were similar, and 13 strains had a higher bacterial load than the Δ*invA* Δ*sseB* strain (Fig. 3E). Similarly, in the mouse model (liver and spleen), 19 (liver) and 18 (spleen) isolates caused lower bacterial load than the Δ*invA* Δ*sseB* strain, 9 (liver) and 10 (spleen) showed no difference compared to the Δ*invA* Δ*sseB* strain, and 8 (liver) and 7 (spleen) isolates had a higher bacterial load (Fig. 3F and G).

10.1128/mSphere.00293-20.2 FIG S2 Bacterial loads and distributions of contrasts in human cells infected with 35 phylogenetically distant *Salmonella* serovars. Bacterial adhesion in epithelial cells (A) and bacterial phagocytosis in macrophages (C) are shown as box plots. Low- and high-virulence controls are shown in green and red, respectively. (B and D) Contrast analysis was used to compare differences in bacterial burden caused by each of the 35 serovars against the low-virulence control Δ*invA* Δ*sseB* strain (mean value shown in green) within all phenotypic traits measured in each model. Epithelial cell adhesion (B) and macrophage phagocytosis (D). Isolate means are shown by black dots. Orange boxes denote the ranges of values, solid black bars show the 95% confidence intervals, and dotted black bars show the confidence intervals when adjusted for multiple testing using Bonferroni’s correction. All isolates whose adjusted confidence intervals do not cross the green line were significantly different from the Δ*invA* Δ*sseB* strain (*P* < 0.05). Either 2, 3, or 4 biological replicates with 3 technical replicates per isolate were used for epithelial cells. Either 2 or 3 biological replicates with 3 technical replicates were used for macrophages. Download FIG S2, PDF file, 0.2 MB. © Crown copyright 2020. 2020 Crown This content is distributed under the terms of the Creative Commons Attribution 4.0 International license.

### Assessment of concordance between *in vitro* and *in vivo* models of *Salmonella* virulence.

**FIG 4:**
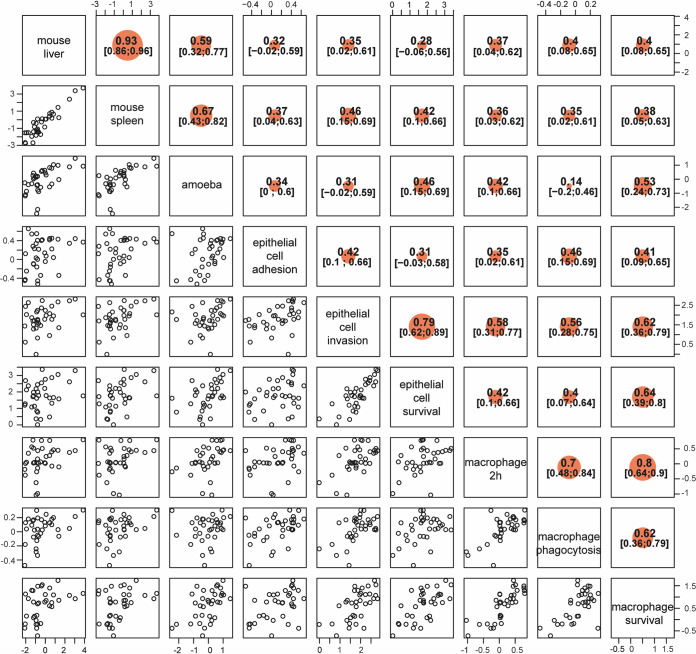
Correlation analysis of 35 strains across the four models of infection. Concordance between each of the four models of infection was assessed by correlation between scaled contrasts from each model. Scattergrams are shown for each of the phenotype combinations on the left of the diagonal running from the top left corner to the bottom right corner. Each point represents a *Salmonella* serovar. Strain positions are dictated by the level of contrast between the bacterial burden (Log_10_ CFUs) caused by an isolate and the bacterial burden caused by low-virulence control Δ*invA* Δ*sseB* strain. At the top, the intraclass correlation coefficient is displayed with 95% confidence interval. Correlation coefficients and confidence intervals are shown to the right of the diagonal. Correlations are significant for all correlation coefficients whose confidence intervals do not include zero. The size of the orange dot reflects the strength of the correlation coefficient.

To compare the different models of *Salmonella* infection in the assessment of the levels of virulence for each isolate, we performed a correlation analysis between contrasts for each model (Fig. 4). All phenotypic traits from each of the models were compared against every other trait. The majority of correlations (86% [31/36]) were positive and significant (Fig. 4). As expected, comparisons that were the most significant were identified within a specific model and included bacterial burden in the mouse spleen versus mouse liver (*r* = 0.93; confidence interval [CI], 0.86 to 0.96), survival in macrophage 2 h p.i. versus 18 h p.i. (*r* = 0.8; CI, 0.64 to 0.9), and epithelial cell invasion versus epithelial cell survival (*r* = 0.79; CI, 0.63 to 0.89). Highly significant correlations were also identified between different models, including bacterial burden in the mouse spleen and liver versus bacterial burden in amoeba (*r* = 0.67 [CI, 0.43 to 0.82] and *r* = 0.59 [CI, 0.32 to 0.77], respectively) and epithelial cell survival and invasion versus macrophages survival (18 h p.i.) (*r* = 0.64 [CI, 0.39 to 0.80] and *r* = 0.62 [CI, 0.36 to 0.79], respectively). The amoeba and mouse models also showed significant correlations (0.42 < *r* < 0.53) with epithelial cell survival and macrophage survival (Fig. 4). Interestingly, the isolates (S3, S5, S33, S40, S51, and S428) with the highest bacterial burden detected *in vivo* in the mouse model of systemic infection were also found to be the highest isolates in the amoeba model and were significantly higher than the low-virulence control in macrophage and epithelial cell models. These isolates with high cellular and tissue burden, with the exception of S51, were all clustered in the phylogenic tree, suggesting shared determinants of virulence (Fig. 1).

### Comparative genomics predict *Salmonella* virulence phenotype.

**FIG 5:**
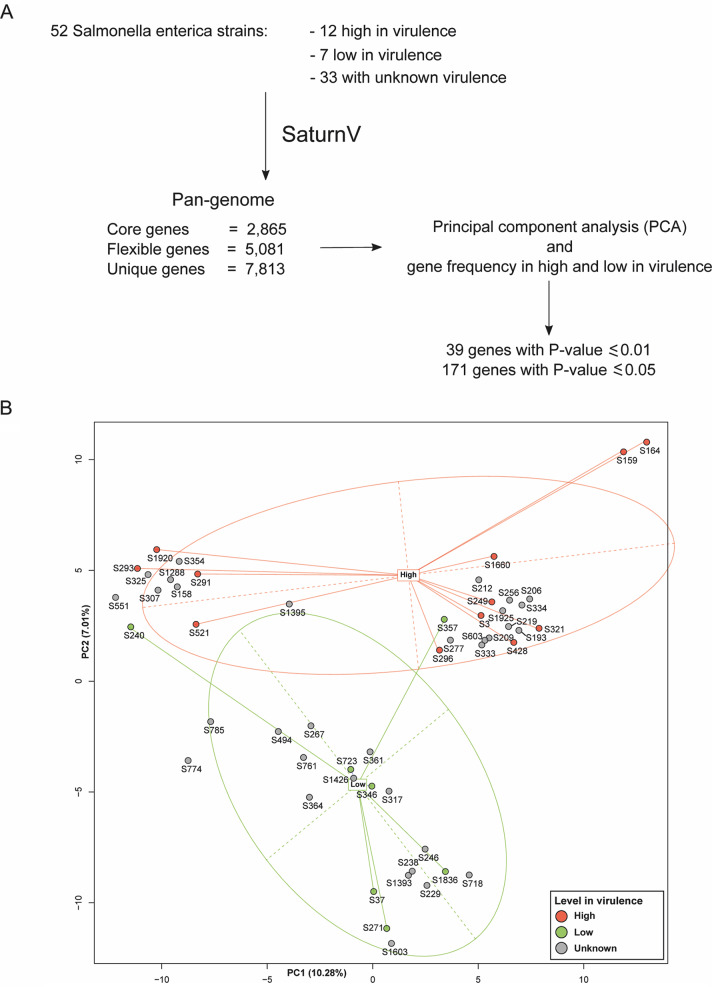
PCA-based prediction model. (A) Workflow of the strategy used to build a PCA-based pan-genome model. The genome sequences for 12 known highly virulent strains, 7 known lowly virulent strains, and 33 strains of unknown virulence were used. (B) PCA plot showing lowly virulent strains in blue, highly virulent strains in red, and strains of unknown virulence in light green. The PCA revealed clustering of genes of similar virulence. Highly virulent strains clustered at the top of the plot, and lowly virulent strains at the bottom. The position of strains of unknown virulence relative to these two groupings was used to predict their phenotype.

Additional isolates were chosen for comparative genomic analyses with the aim of identifying virulence-associated genes. These isolates were phenotyped in both the epithelial cell model and the mouse model. Nineteen strains showing a concordant phenotype in both models, 12 strains with a high level of associated bacteria and 7 with a reduced number of bacteria, were selected for comparative genomic analyses (Table 2). The pan-genomes of 52 S. enterica genomes were analyzed using SaturnV (v1.1.0), including 19 isolates with known levels of virulence and 33 isolates untested in the models of infection, and were used to benchmark the prediction of the number of *Salmonella-*associated levels as high or low. The presence/absence of flexible genes was compared by principal-component analysis (PCA) (Fig. 5A). Among the flexible genes, we identified 171 genes that were significantly (*P* ≤ 0.05) correlated with the predicted phenotype (see Table S1). The resulting PCA plot revealed that strains appeared to segregate based on their virulence phenotype. Isolates with high burden demonstrated a tendency to cluster, and likewise, a low-burden cluster was clearly evident. These data clearly showed a link between the level-of-virulence (high or low) phenotype in an isolate and gene content.

**TABLE 2:** *Salmonella* isolates with high- and low-virulence phenotypes used for pan-genomic analysis

Isolate ID	Serotype	Source category	Host organism	Specific source	Virulence
S3	Enteritidis	Human	Human		High
S51	Tyresoe	Fish/shellfish	Shrimp		High
S164	Typhimurium	Animal other			High
S249	Virchow	Fish/shellfish	Shrimp		High
S291	Brandenburg	Fish/shellfish	Clams		High
S293	Brandenburg	Dairy		Raw milk	High
S296	Hvittingfoss	Fish/shellfish	Shrimp		High
S321	Blockley	Poultry	Chicken	Heart	High
S428	Heidelberg	Human	Human	Feces	High
S521		Environmental			High
S1660	Paratyphi B	Fish/shellfish	Shrimp		High
S1920	Montevideo	Nuts/seeds	Almond	Almond kernels, raw	High
S37	Falkensee	Other		Rice seasoning	Low
S240	Montevideo	Nuts/seeds	Sesame	Sesame, halva, pistachio	Low
S271	Senftenberg	Nuts/seeds	Sesame	Tahini	Low
S346	Liverpool	Plant (fruit/veg)	Cantaloupe		Low
S357	London	Fish/shellfish	Shrimp		Low
S723	Enteritidis	Unknown			Low
S1836	Kentucky	Nuts/seeds	Almond	Almond kernels, raw	Low

10.1128/mSphere.00293-20.5 TABLE S1 List of the 171 proteins that correlated (*P* value ≤ 0.05) with Salmonella enterica isolate virulence. Corresponding genes and positions on *Salmonella* Typhimurium SL1344 chromosome as well as the protein functions based on UniProt, Virulence Factor DataBase (VFDB), and BacMet databases are included. Download Table S1, XLSX file, 0.1 MB. © Crown copyright 2020. 2020 Crown This content is distributed under the terms of the Creative Commons Attribution 4.0 International license.

Given that the association between genotype and gene content was represented via the PCA plot, we hypothesized that the PCA could be used to predict the virulence of untested *Salmonella* isolates used as a benchmark in predicting high and low levels of *Salmonella* virulence without *a priori* knowledge of the *in vitro* and *in vivo* screening strategies described above. The pan-genomes of 33 isolates of unknown levels of virulence were included in pan-genome analysis and added to the PCA (Table 3, Fig. 5B). The positions of the 33 strains within the PCA plot were used to predict their potential virulence level, those clustering with strains with known high burden were predicted to be high in virulence while those clustering with strains with low burden were predicted to be low in virulence. As a result, 15 strains were predicted to be low and 18 were predicted to be high (Fig. 5B). This group of strains of unknown virulence was then tested in the epithelial cell and the mouse models of infection to assess this prediction and define the levels of accuracy.

**TABLE 3:** *Salmonella* strains with virulence phenotypes predicted by PCA

Isolate ID	Serotype	Source category	Host organism	Specific source	PCA prediction
S158		Plant (fruit/veg)	Blueberry		High
S193	Thompson	Plant (fruit/veg)	Spinach		High
S206	Muenchen	Plant (fruit/veg)	Cantaloupe		High
S209	Braenderup	Poultry		Egg: liquid, whole	High
S212	Stanley	Other		Spices: black pepper	High
S219	Hadar	Poultry	Chicken	Rinse	High
S229	Derby	Animal/other	Pork	Muscle	Low
S238	Mbandaka	Other		Pasta	Low
S246	Kentucky	Other		Animal feed meat and bone meal	Low
S256	Bovismorbificans	Animal/other	Beef		High
S267	Kiambu	Poultry	Chicken	Livers	Low
S277	Uganda	Animal/other	Beef	Ground	High
S307	Poona	Plant (fruit/veg)	Cantaloupe		High
S317	Ohio	Plant (fruit/veg)	Cocoa	Beans	Low
S325	Bredeney	Dairy		Goat cheese	High
S333	Berta	Other		Cheese	High
S334	Kottbus	Poultry	Turkey		High
S354	Gaminara	Other		Spices: coriander	High
S361	Adelaide	Animal/other	Beef	Roast	Low
S364	Cerro	Poultry		Egg: liquid frozen, albumin	Low
S494		Environmental			Low
S551	r:e,n,x	Environmental		Multistate *Salmonella* in cantaloupe outbreak	High
S603	Bareilly	Environmental		Multistate *Salmonella* in cantaloupe outbreak	High
S718	Derby	Poultry	Turkey		Low
S761	Stanleyville	Unknown			Low
S774	58:d:z6	Human	Human		Low
S785	11:b:e,n,x	Unknown			Low
S1288	Javiana	Plant (fruit/veg)	Soy	Soya spruce	High
S1393	Tornow	Nuts/seeds	Peanut		Low
S1395	G(1):b,−	Nuts/seeds	Peanut		High
S1426	Berta	Environmental		Laurel Creek	Low
S1603	Havana	Animal/other	Porcine	Cecal content	Low
S1925	Muenchen	Nuts/seeds	Almond	Almond kernel, raw nonpareil variety	High

**FIG 6:**
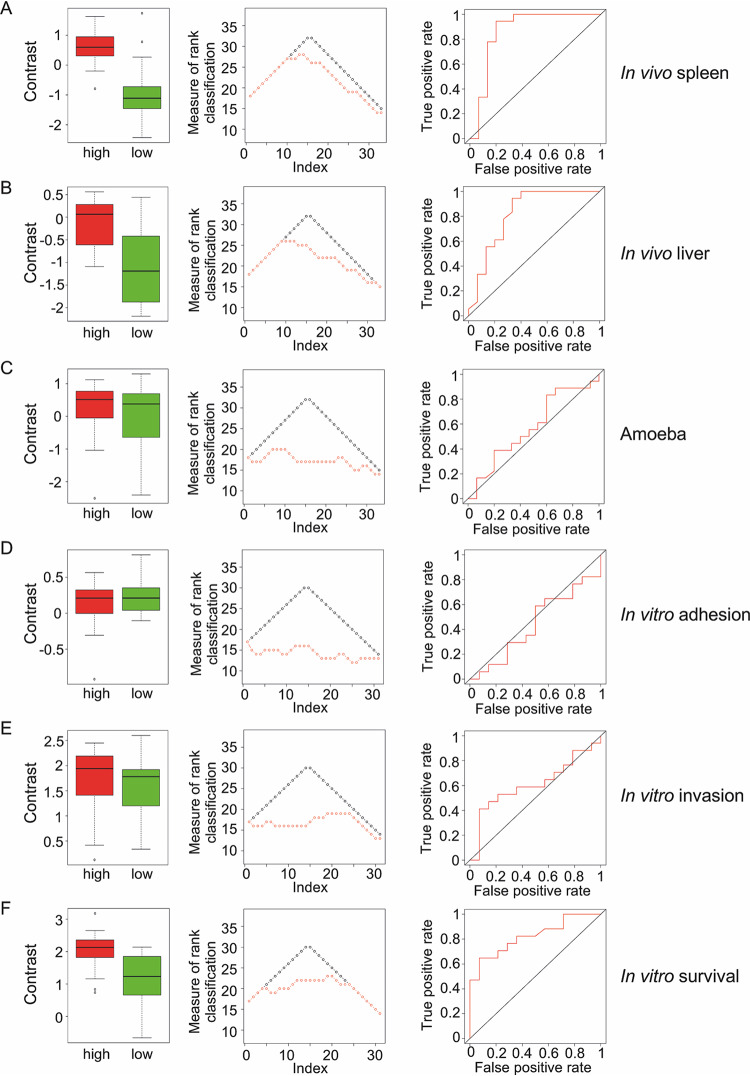
Concordance analysis to test virulence prediction. (A to E) Distributions of contrasts in low- and high-prediction groups (left), measure of rank classification (middle) and ROC curves (right) for the different models. The distribution of contrast values in each group (predictions, low and high) was evaluated using *t* test and Mann-Whitney Wilcoxon test. The variance explained (*R*^2^) was calculated using ANOVA with one factor (prediction group: low or high). Percentage of agreement compared the groups (low versus high) from the prediction and the groups (low versus high) from the observations based on contrast. For the measure of rank classification, perfect prediction is shown in black and observed prediction in red.

As described previously, contrast analysis was performed to classify the 33 strains based on the resulting bacterial burdens relative to the low-virulent Δ*invA* Δ*sseB* strain for all phenotypic traits tested (see Fig. S3). The distributions of contrast values in the predicted groups (low and high) for each observed phenotypic trait are shown as boxplots (Fig. 6, left). The difference between the benchmarked predictions and expected rank was used as a measure of rank classification (Fig. 6, middle). We also used the area under the curve (AUC) of the receiver operating characteristic (ROC) as a predictive measure, with higher values showing better prediction (Fig. 6, right). Other measurements of concordance included variance explained (*R*^2^) and percentage of agreement within each group (low and high) for the observations based on contrasts (Table 4). The phenotypic traits showing significant concordance with predicted virulence were *in vivo* systemic infection in mice (82% of agreement for the spleen and 76% for the liver) and *Salmonella* survival in epithelial cells (74% agreement) (Fig. 6 and Table 4). Taken together, these data showed the predictive value of pan-genome analysis.

**TABLE 4:** Model comparisons for *Salmonella* serovar virulence prediction

Model	*t* test	Wilcoxon test	*R*^2^	% agreement	Kappa coefficient	Mean rank difference	Area under the curve
*In vivo* infection							
Spleen	1.3e−4	3.7e−4	0.45	0.82	0.63	2.18	0.87
Liver	9.6e−4	1.8e−3	0.34	0.76	0.51	2.85	0.82
Epithelial cell							
Adhesion	0.37	0.6	0.03	0.55	0.09	8.55	0.44
Invasion	0.52	0.31	0.01	0.55	0.09	5.97	0.61
Survival	3.9e−3	2.6e−3	0.27	0.74	0.48	2.77	0.82

10.1128/mSphere.00293-20.3 FIG S3 Distributions of contrasts in the four models of infection for *Salmonella* strains with virulence phenotypes predicted by PCA. Contrast analysis was used to compare differences in bacterial burden caused by each of the 33 serovars against the low-virulence control Δ*invA* Δ*sseB* strains (mean value shown in green) within defined phenotypic traits measured in each model. Epithelial cell adhesion (A), invasion (B), and survival (C), replication in amoeba (D), and bacterial loads in mouse spleen (E) and liver (F). Isolate means are shown by black dots. Orange boxes denote the ranges of values, solid black bars show the 95% confidence intervals, and dotted black bars show the confidence intervals when adjusted for multiple testing using Bonferroni’s correction. All isolates whose adjusted confidence intervals do not cross the green line were significantly different from the Δ*invA* Δ*sseB* strain (*P* < 0.05). Either 2, 3, or 4 biological replicates with 3 technical replicates per isolate were used for epithelial cells. Three biological replicates were performed per isolate in the amoeba. Six biological replicates were performed per isolate in the mouse. Download FIG S3, PDF file, 0.2 MB. © Crown copyright 2020. 2020 Crown This content is distributed under the terms of the Creative Commons Attribution 4.0 International license.

### Levels of *Salmonella* isolate virulence are associated with the presence or absence of new virulence genes.

**FIG 7:**
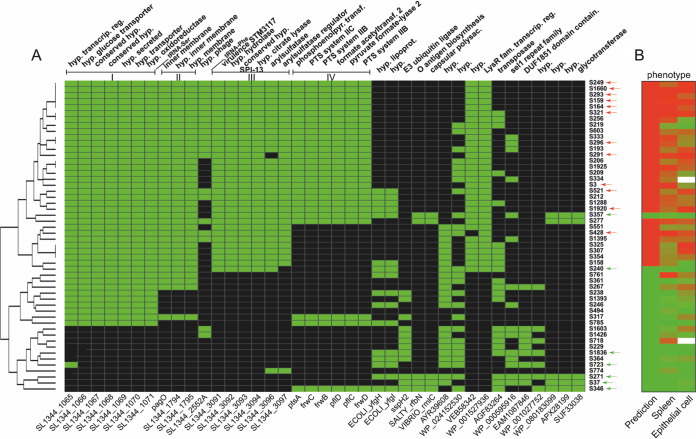
Distribution of virulence genes in *Salmonella* isolates used in the PCA-based prediction model. (A) Heat map showing the distribution of 39 genes correlating with the levels of virulence. Column labels indicate gene names (bottom) and functions (top). The gene names at the bottom refer either to the genes in *Salmonella* Typhimurium SL1344 or, when no annotation was available, to a generic name generated by Prokka (linked to Table S1 in the supplemental material). The lines above mark contiguous genes on the *S.* Typhimurium SL1344 chromosome and show four clusters (I to IV). Green cells indicate that the gene is present, whereas black cells indicate its absence from a given isolate. The tree on the left shows isolates ordered based on hierarchical clustering. The identifiers (IDs) of the isolates are shown on the right. Red arrows indicate high-virulence isolates, and green arrows indicate low-virulence isolates that were used for PCA analysis. hyp, hypothetical; transcrip, transcriptional; reg, regulator; phosphoenolpyr transf, phosphoenolpyruvate transferase; acetyltransf, acetyltransferase; lipoprot, lipoprotein; polysac, polysaccharide; fam, family. (B) Heat map showing the distribution of predicted phenotype and phenotype *in vivo* (spleen) and *in vitro* (epithelial cell survival). Isolates were ranked according to the distribution of contrasts. Red cells, high virulence; green cells, low virulence. Isolates S324 and S718 (empty cells) were gentamicin-resistant and not tested *in vitro*.

We analyzed the genetic diversity and population structure of *Salmonella* isolates used in the PCA analysis. We performed hierarchical clustering for the 39 most significant genes (*P* ≤ 0.01) present or absent in the 19 *Salmonella* isolates with known virulence and visualized the 33 isolates with predicted levels of virulence (Fig. 7). Of major interest in Fig. 7 was the identification of 4 major clusters, including SPI-13 and a collection of 21 hypothetical and conserved hypothetical proteins which may play a significant role in virulence. The low-virulence phenotype appeared to be conferred by the absence of several clusters of genes encoding proteins for metabolic functions or virulence factors. Cluster I is delimited by the serine tRNA and encodes a putative sialic acid transport system, a member of a sodium solute symporter family. Sialic acid is involved in several biological process and can be used as a nutrient source. This system was shown to be upregulated under anaerobic conditions (21) and to be a reversible carrier, a propriety of sialic acid transporters associated with bacteria that colonize humans (22). Cluster II encodes 3 putative membrane proteins, including *pagO*, a PhoP-activated gene (23). In *S.* Typhimurium, this cluster was found to be upregulated when grown under an SPI-2-inducing condition (21) and in both macrophages and HeLa cells (24). These 3 genes were also found to be regulated by RcsB (25), a two-component system involved in virulence, cell envelope stress, antimicrobial resistance, and oxidative and acidic stress (26–28). Cluster III encodes the first 6 genes of SPI-13 (2 operons), which were shown to be involved in systemic infection of mice (29). Open reading frame (ORF) STM3117 is involved in methylglyoxal detoxification and is known to promote the replication of *Salmonella* Typhimurium inside host macrophages (30) and epithelial cells (31). The first 4 genes were upregulated in macrophages but not in HeLa cells (24). Cluster IV encodes a putative sugar-transporting phosphotransferase system (PTS) that maybe involved in anaerobic carbon metabolism (32). This cluster was upregulated during anaerobic growth (21). In *Klebsiella*, the *frw* system was involved in virulence in mice (33). Three hypothetical and uncharacterized proteins, including a putative regulator (WP_001527936), were associated with the isolates with the highest bacterial burden.

Within the isolates used to build the PCA, there were 2 low-virulence isolates (S357 and S240) that segregated with the cluster with high virulence (Fig. 5B and Fig. 7). When we compared the pan-genome of S357 to those of the 12 selected strains with high bacterial loads, we detected an indel (deletion of a T) at the 6th nucleotide position in *ssaK* resulting in a frameshift and giving a truncated protein (see Fig. S4A). This indel affecting the function of SsaK may have a major impact on the level of virulence of S357, because SsaK is known to be an essential component of the SPI-2 secretion system apparatus of *S.* Typhimurium (34). SsaK was described as a cytoplasmic protein interacting with SsaN forming the C ring complex in the SPI-2 type 3 secretion system (T3SS), an essential part of a 13-gene transcriptional unit of 10,161 nucleotides from *ssaK* to *ssaU* (35–37). Moreover, mutants in the *ssaK-ssaU* operon were shown to invade fewer epithelial cells by reducing SPI-1 secretion (34).

10.1128/mSphere.00293-20.4 FIG S4 Genomic alterations in isolates S357 London and S240 Montevideo. (A) Graphical representation of the indel in the *ssaK* gene of S357 causing a frameshift and resulting in a premature stop codon. The slash bars represent truncated regions for better visualization of the key regions. The putative *ssaK* ORF in the S357 genome begins 102 nucleotides (nt) downstream of the premature stop codon and encodes a putative 537-amino-acid protein instead of the expected 675-amino-acid SsaK protein in SL1344. (B) Genomic deletion of 75.2 kbp in the S240 isolate was identified between *hycE* (start position on SL1344, 3,017,328 bp) and *truD* genes (end position on SL1344, 3,092,532 bp) and includes the SPI-1 T3SS essential for *Salmonella* virulence. (C) *invA* deletion impaired the virulence of *Salmonella* Typhimurium *in vivo* as shown by reduced bacterial recovery in spleen and liver and low competitive index (CI). C57BL/6J mice were coinfected with a mixed inoculum containing equal numbers of SL1344 and *inv*A-deficient SL1344 cells. CIs were calculated by dividing the number of cells for the Δ*invA* strain (kanamycin resistant) by that for SL1344 (streptomycin resistant). Download FIG S4, PDF file, 0.2 MB. © Crown copyright 2020. 2020 Crown This content is distributed under the terms of the Creative Commons Attribution 4.0 International license.

We also noted the S240 isolate clustered with the high-virulence group, contradicting the *in vitro* and *in vivo* analysis showing that S240 has a low-virulence phenotype. The low virulence of S240 is presumably caused by a deletion (between the 3′ end of *hycE* and the 5′ end of *truD*) of 73 genes located within a 75.2-kb region encompassing SPI-1 and including *invA* (Fig. S4B). Consistent with this deletion reported previously by others, the loss of *invA* diminished the virulence of SL1344 *in vivo* (Fig. S4C).

It is possible that strains S240 and S357 have evolved by genome degradation to adapt to their host (nut and shrimp) and lost their virulence potential in the mammalian host. Gene decay was linked with *Salmonella* host specialization (38).

For all the isolates with predicted levels of virulence, only 3 of 33 isolates had *in vivo* and/or *in vitro* phenotypes that did not match the prediction: S219 was predicted high but presented low virulence *in vivo* and *in vitro*, and isolates and S718 and S761 were predicted as low virulence but were high. In S219, there were nine altered genes (*yebT*, *phsA*, *yegD*, *queG*, *basR*, *bcsQ*, *nifJ*, *osmX*, and *yadI*), 4 with IS*6* insertions, 4 with premature stop codons, and 1 missing. The high number of IS*6* insertions in isolate S219 may render this strain of serovar Hadar less virulent. Strain S718 belongs to serovar Derby, and we have not identified why this isolate was virulent. For S761, phylogenetic analysis of the SalFoS strains indicated that this isolate of serovar Stanleyville shares a recent common ancestor with several *S.* Typhi and *S.* Paratyphi A strains (data not shown).

One major observation from these detailed combined *in vitro*, *in vivo*, and genomics-based analyses is the identification of several novel genes and clusters associated with virulence.

## DISCUSSION

The systematic approach of high-quality WGS coupled with comparative genomics analysis is a powerful strategy to determine the phylogenetic relationships between Salmonella enterica isolates (39, 40). Combining *in vitro* and *in vivo* systematic analysis to define isolates as high and low in virulence with the presence and absence of genes clearly showed a significant correlation for most isolates. In addition, WGS-derived phylogenetic analysis is an invaluable tool in epidemiological investigations and has enabled fast and accurate identification of the sources of foodborne disease outbreaks (41–43). Other studies have used WGS to characterize *Salmonella* serovars for antimicrobial resistance and virulence genes using collections of human, animal, and environmental isolates (44–46). However, virulence prediction was mainly based on already-known virulence genes, and only one phenotypic model was tested.

Here, we hypothesized that the levels of virulence, as measured by the *Salmonella* burden in cells and tissues using different models of infection, may be predicted as high or low when combining WGS, phylogeny, and pan-genome studies for the presence and absence of genes in association with virulence. As additional supporting evidence, we benchmarked a collection of 33 isolates with no *a priori* knowledge of their levels of virulence, and which remarkably supported the potential of this approach. We entertain the possibility that this is a first step toward new ways of identifying other genes implicated in virulence, as we identified here a collection of 20 hypothetical and conserved hypothetical proteins. Additional studies will be essential to clearly define the role of these unknown genes in virulence. As most of these genes are involved in metabolism, they may represent genes important for nutrient acquisition within the host. We propose that gaining a clear understanding of the genetic determinants causing virulence in different *in vitro* and *in vivo* models of infection will enable pathogenic potential to be predicted by WGS analysis. We have used WGS to build associations between phenotypic and genetic diversity within 83 isolates representing 77 different Salmonella enterica serovars. *Salmonella* isolates were selected to maximize genetic diversity, and accordingly, we observed a wide range of phenotypes, with the numbers of bacteria spanning beyond the ranges of the high- and low-virulence controls. Interestingly, the magnitudes of the differences between the controls varied between the different models tested. Even if the difference between the wild type (WT) and the Δ*invA* Δ*sseB* strain was small in the macrophage and amoeba models, the distribution of phenotypes was broad in these models. The correlation between the different models of infection (epithelial cell, macrophage, amoeba, and mouse models) in assessing virulence was highly significant. The isolates with the highest burden were found to be high in all models. The isolates that did not replicate well either in epithelial cells, in macrophages, or in amoeba were the least virulent in the mouse model. Some serovars replicated at high levels in epithelial cells and macrophages but showed low virulence in the amoeba and/or mouse models, highlighting the fact that a high propensity for adhesion to, invasion of, or survival in epithelial cells does not guarantee that a strain is pathogenic at the level of the whole organism. On the other hand, *Salmonella* needs to be able to replicate in epithelial cells and/or macrophages to cause disease, showing the importance of studying the biodiversity of *Salmonella* isolates in various models of infection.

Our analysis showed that the virulence of clinical isolates of different serovars (Hartford, Enteritidis, Heidelberg, Newport, Bareilly, and r:e,n,x) was consistently high in all *Salmonella* infection models (*in vitro* and *in vivo*), highlighting the validity of using modeling in cells and animals to identify highly virulent isolates from different serovars. Other highly virulent serovars were isolated from fish/shellfish (Tyresoe, Cremieu, and Hull), from meat and animal products (Derby, Uganda, and Berta), and from plant/fruits/vegetables/nuts (Javiana, Stanley, and Muenchen). *Salmonella* isolates within one serovar did not necessarily present the same degree of virulence as observed in the present study for the serovars Derby, Enteritidis, and Montevideo, stressing the potentially high genomic variability not only between serovars but also within one individual serovar. Hence, this shows that WGS coupled to other genomics approaches has an excellent potential in studying virulence in foodborne disease outbreaks.

Analysis of genetic relatedness among highly and poorly virulent *Salmonella* serovars was based on pan-genome analysis where the pool of flexible genes was used to identify gene frequency for high- and low-virulence serovars. The predictive value of the PCA-based model was high in the model of epithelial cell infection (74% concordance) and in the systemic infection mouse model (82% concordance). It is clear that a gene-based frequency approach to determine virulence has some limitations. In fact, there were 3 serovars (S219, S718, and S761) that did not fit the prediction, contradicting the *in vitro* and *in vivo* phenotypes. False negatives are a major concern, as they pose the greatest public health risk.

Genetic variability between serovars occurs for a variety of reasons, such as point mutations and single nucleotide insertions or deletions; additionally, there is a role of unknown genes in virulence. Further refinement may be improved by using a larger number of serovars with known phenotypes to predict virulence together with an approach that weighs in nucleotide variants and kmers. These studies will certainly assist in integrating WGS approaches into food safety practices and prediction of outbreaks.

## MATERIALS AND METHODS

### *Salmonella* strains.

*Salmonella* strains used in this study were selected from SalFoS (The Salmonella Foodborne Sys-OMICS) database, a repository of thousands of *Salmonella* isolates from environmental, plant, animal, food or clinical sources (https://salfos.ibis.ulaval.ca/). *Salmonella* strains used in this study are listed in Tables 1 to 3. *Salmonella* serovars rarely implicated in foodborne disease in Canada were selected and compared to clinical and foodborne isolates of Salmonella Enteritidis, *Salmonella* Heidelberg, and *Salmonella* Typhimurium, all of which are among the top ten serovars that commonly cause salmonellosis in Canada. The rationale for including isolates from outside the top 70 salmonellosis-causing serovars was based on the hypothesis that since these isolates do not cause many salmonellosis cases, they were likely to be less virulent. The high-virulence control was *Salmonella* Typhimurium SL1344, and the isogenic Δ*invA* Δ*sseB* mutant was used as the low-virulence control. All the bacteria used in this study had similar growth curves to that of the high-virulence control in LB.

### *In vitro* human cell studies.

**(i) Interactions with human epithelial intestinal cells.** A high-throughput gentamicin protection assay was adapted for 96-well plates to quantify *Salmonella* interactions with human intestinal epithelial cells (47). INT-407 cells (ATCC CCL-6) were grown in minimal essential medium (MEM) supplemented with 10% heat-inactivated fetal bovine serum (FBS) and 25 mM HEPES (Wisent). Bacteria were grown overnight under static conditions (low aeration) in LB-NaCl (300 mM) to induce SPI-1 and were added at a multiplicity of infection of 20:1 in triplicates. After 90 min, infected cells were washed with phosphate-buffered saline (PBS), and fresh medium supplemented with 50 μg/ml gentamicin sulfate was added to kill the extracellular bacteria. Cells were lysed with PBS-deoxycholate (DOC) 0.1% at 90 min (adhesion), 180 min (invasion), and 18 h (survival) postinfection. Serial dilutions were performed for enumeration of viable colony counts (CFU/ml). Sample size varied across isolates, ranging from 2 to 4 biological replicates, with 3 technical replicates per biological replicate.

**(ii) Interactions with human macrophages.** THP-1 cells (ATCC TIB-202) were maintained in RPMI 1640 supplemented with 10% heat-inactivated FBS, 1 mM sodium pyruvate, and 1% MEM nonessential amino acids (Wisent). The monocyte cells were differentiated into macrophages by addition of 10^−7^ M phorbol 12-myristate 13 acetate (PMA) (Sigma) for 48 h before the infection. Following an overnight growth in LB broth, the strains were added in triplicates at a multiplicity of infection of 10:1. After 30 min, infected cells were washed with PBS, treated with gentamicin (50 μg/ml), and lysed with PBS-DOC 0.1% at 30 min (phagocytosis), 2 h, and 18 h (survival) postinfection; then, serial dilutions were performed for enumeration of viable colony counts (CFU/ml). Either 2 or 3 biological replicates were performed per isolate, with 3 technical replicates per biological replicate. Control SL1344 and Δ*invA* Δ*sseB* strains each had a total of 6 biological replicates per phenotypic trait.

### Amoeba studies.

**(i) Amoebic cells and culture conditions.** The strain of *Acanthamoeba* used in this study was A. rhysodes (ATCC 50368). It was obtained as an axenic strain and maintained as adherent cells in an axenic culture medium, peptone-yeast extract-glucose (PYG) broth, in 25-cm^3^ tissue cultures flasks incubated at 30°C until near confluence was reached. Amoeba suspensions were examined by bright-field microscopy before use, and the number of cells was determined by counting with a Neubauer chamber.

**(ii) Preparation of bacterial inocula.** *Salmonella* strains were grown in LB-NaCl (300 mM) at 37°C until late-logarithmic growth phase to promote induction of SPI-1. The bacteria were then washed twice and diluted in PBS to a concentration of 100 bacteria to 1 amoeba.

**(iii) Intracellular bacterial growth in *A. rhysodes*.** *A. rhysodes* was grown in 10 ml of PYG broth. The tissue culture flask containing near confluent *A. rhysodes* was gently shaken, and the PYG medium containing nonadherent amoeba was removed. Adherent amoebae were removed by agitating the empty flask and resuspending with fresh PYG broth. The suspension was seeded into a 12-well plate (10^5^ cells/well). Amoeba were then incubated for 24 h at 30°C to permit adherence to the wells. The number of amoebae per well was calculated an additional time prior to infection. To prepare *Salmonella* inocula, bacteria were diluted in PBS to give a multiplicity of infection of 100. Following inoculation, the 12-well plate was centrifuged for 5 min at 500 × *g* to promote contact between bacteria and amoebae. The plate was then incubated at 30°C for 60 min. After incubation, the medium was removed, wells were washed with PBS, and fresh PYG broth supplemented with 50 μg/ml gentamicin sulfate was added to kill the extracellular bacteria. After incubation for 30 min at 30°C, the wells were washed twice with PBS and lysed in 0.5% sodium deoxycholate. CFU enumeration was then determined by serially diluting the samples and plating them on LB agar. Three biological replicates were performed per isolate. Control SL1344 and Δ*invA* Δ*sseB* strains were replicated a total of 51 times.

### Mouse studies.

**(i) Animals.** All animal experiments were performed under guidelines specified by the Canadian Council for Animal Care. The animal use protocol was approved by the McGill University Facility Animal Care Committee (protocol no. 7760). C57BL/6J mice were obtained from The Jackson Laboratory (Bar Harbor, ME, USA) and then bred in-house.

**(ii) *In vivo Salmonella* infection.** For intravenous (i.v.) infection, bacteria were grown shaking at 37°C in tryptic soy broth (TSB) to an optical density at 660 nm (OD_660_) of between 0.1 and 0.2, cooled to 4°C, and plated overnight on tryptic soy agar. The day after, the infectious dose was adjusted to 500 CFU/μl with sterile saline, and 200 μl was injected into each of the caudal veins of 7- to 13-week-old mice of both sexes. For oral infections, bacteria were grown overnight in 3 ml of TSB shaking at 37°C. Mice were infected by oral gavage with 200 μl of a 10-fold dilution of the overnight culture containing 2 × 10^7^ to 3 × 10^7^ CFU of *Salmonella* spp. The infectious doses were verified by the plating of serial dilutions on Trypticase soy agar (48, 49). Mice infected orally were first deprived of food for 4 h before infection with Salmonella enterica. Spleens, livers, and feces were collected day 3 postinfection and homogenized in saline, and CFUs were determined by plating serial dilutions on either Trypticase soy agar (spleen and liver) or MacConkey (feces) plates. A sample size of 6 mice was used per isolate, 3 female and 3 male mice, with the exception of S3 (4 mice), S5 (5 mice), S32 (5 mice), S428 (5 mice), and S334 (4 mice). Three mice were tested per control for each experiment. Across all experiments, SL1344 was replicated 57 times and the Δ*invA* Δ*sseB* strain was replicated 48 times.

### Statistical analyses.

**(i) Comparing virulence levels.** The bacterial burden, or colony counts, found in each of the 35 serovars was compared to that of the low-virulence control by analysis of variance (ANOVA). Models were fit separately for each of the four models:

*(a) Infection of human epithelial cells*. The data consist of CFU counts for 35 serovars and the low-virulence control, under 3 conditions (bacterial adhesion, invasion, and survival), and for several epithelial cells per serovar. There were 2 to 3 replicates per combination, leading to an unbalanced study design containing 840 measurements. We fit the following model that contains a random effect accounting for dependence between results from the same cell (equation 1):

(1) $$y_{\mathrm{ijkl}}=S_{j}+C_{k}+S_{j}\times C_{k}+b_{i}+e_{\mathrm{ijkl}}$$

where *y_ijkl_* is the CFU counts (in log_10_ units) measured in the *i*th cell from serovar *j* under condition *k*, replicate *l*; *S_j_* is the serovar *j*, *C_k_* is the condition *k*; *b_i_* is the random term associated with the cell *i*. Finally, *e_ijkl_* represents the normally distributed error term of the statistical model. This statistical model is equivalent to a two-way ANOVA for serovar and condition, with the additional random term for cells. Contrasts compared serovars to the low-virulence control strain.

*(b) Human macrophages*. The data consist of 731 measures of CFU counts across the serovars and three conditions (uptake, survival 2 h pos infection, and survival at 18 h postinfection). As for the epithelial cells, an ANOVA model was fit to log CFU counts in this unbalanced design, including terms for serovar, condition, and their interaction, and again including a random term to account for intracell correlations. The model is similar to that in equation 1.

*(c) Amoeba model*. The data consist of 147 measures of CFU counts. The bacterial burden was modeled as a function of serovar with a one-way ANOVA analysis as follows (equation 2):

(2) $$y_{\mathrm{ij}}=S_{j}+e_{\mathrm{ij}}$$

where *y_ij_* is the CFU counts (in log_10_ units) measured in the *i*th cell from serovar *S_j_*; *e_ij_* represents the normally distributed error term of the statistical model.

*(d) Mouse model*. The data consist of 514 CFU counts. We modeled the bacterial burden as a function of the serovar and the organ (called the condition: liver and spleen) as well as their interaction. A random term was added to account for the intramouse correlation, as each mouse provided two observations, one from each organ. The model (equation 3) is as follows:

(3) $$y_{\mathrm{ijk}}=S_{j}+C_{k}+S_{j}\times C_{k}+b_{i}+e_{\mathrm{ijk}}$$

where *y_ijk_* is the CFU counts (in log_10_) measured in the *i*th mouse from serovar *S_j_* under condition *k*; *C_k_* is the condition or organ *k*; *b_i_* is the random term associated with mouse *i*. Finally, *e_ijk_* represents the normally distributed error term of the statistical model. This statistical model is equivalent to a two-way ANOVA with a random effect for mouse.

For each of the four models, type III tests of the hypothesis were computed to evaluate the significance of the serovar-condition interaction (except for experiment 3, where the effect of the serovar was tested directly). Contrasts were constructed to evaluate the difference in log_10_ CFU counts between each of the 35 serovars and the low-virulence control for each condition—i.e., for each phenotypic trait—yielding 70 comparisons all together. Test results and confidence intervals obtained for each contrast were adjusted for multiple comparisons using Bonferroni’s correction. Analyses were performed using R software. The nlme package was used for random effect models and the multcomp package was used for multiple comparisons.

**(ii) Concordance analysis.** Pairwise Pearson’s correlation coefficients were calculated between the estimated contrast values, resulting from the ANOVAs, for each condition (phenotypic trait) and each model.

**(iii) Performance of predictions on new strains.** To assess the accuracy of the predictions of virulence obtained from the PCA analysis (high versus low virulence), 33 new strains with unknown virulence were examined. First, we repeated the analysis similar to that described above (“Comparing virulence levels”) for log CFU counts for each of the 33 new strains compared to low-virulence controls for each phenotypic trait and for three of the four infection models (mouse, amoeba, and epithelial). Contrasts were obtained as described above. Then, the resulting contrasts were compared to predictions from the PCA analysis. A prediction would be defined as perfect if the largest contrast value among the group of strains predicted as low virulence is smaller than the smallest contrast value among the group of strains predicted as high virulence. Performance of the predictions was assessed in five different ways.

1. Boxplots show the distributions of estimated contrasts in each predicted group. The distributions were compared with *t* tests and Mann-Whitney tests. Larger differences imply better predictions.
2. One-way ANOVA models were fit predicting the contrasts as a function of the predicted group. Larger coefficients of determination ($R^{2}$) mean a better prediction.
3. Contrast values were dichotomized into low versus high while matching the numbers of strains in the predicted low and high groups. Accuracy was calculated as the percentage of strains given the same label and also using a Kappa coefficient for concordance between two binary variables. Larger Kappa values imply better agreement.
4. For measures of rank classification, strains were ranked by their contrast values. For each rank, we counted the number of strains in the predicted low-virulence group among all strains with lower rank, and correspondingly, we counted the number of strains predicted to be in the high-virulence group among all strains with higher rank. These counts were plotted together with counts expected under perfect predictions, and we averaged the absolute value of the difference between these counts. A lower value means a better prediction.
5. The receiver operating characteristic (ROC) curves and areas under the curves (AUCs) were calculated. A higher AUC means a better prediction. The *P* value associated with the AUC corresponds to the test of the null hypothesis H0: AUC = 0.5 corresponding to a random prediction.

### Genomic analyses.

**(i) DNA sequencing.** Genomic DNA was extracted from overnight LB broth cultures at 37°C using the E-Z 96 tissue DNA kit (Omega Bio-Tek, Norcross, GA, USA). Around 500 ng of genomic DNA was mechanically fragmented during 40 s by a Covaris M220 (Covaris, Woburn, MA, USA) using the default settings. Libraries were synthesized using an NEBNext Ultra II DNA library prep kit for Illumina (New England BioLabs, Ipswich, MA, USA) according to manufacturer’s instructions and were sequenced by an Illumina MiSeq 300-bp paired-end run at the Plateforme d’Analyses Génomiques of the Institut de Biologie Intégrative et des Systèmes (Laval University, QC, Canada).

**(ii) Core SNP phylogeny.** Core genes of the distant *Salmonella* isolates were determined by SaturnV (v1.1.0) (https://github.com/ejfresch/saturnV) (18) with ≥50% identity between proteins and ≥85% alignment coverage. To avoid bias in the phylogenetic analysis caused by duplicated genes (paralogs), single-copy core genes were retained and aligned using MUSCLE (v3.8.31) (50). Uninformative SNPs were removed by BMGE (v1.12) (51), and the phylogenetic tree was computed by FastTree (v.2.1.9) (52).

**(iii) Pan-genome analysis.** Pan-genome analysis of 52 *Salmonella* strains, of which 12 were highly virulent, 7 were lowly virulent, and 33 strains had an unknown virulence phenotype, was performed using SaturnV (v1.1.0) with ≥90% identity between proteins and ≥85% alignment covering their length to be considered ortholog proteins.

**(iv) Principal-component analysis.** The table of ortholog proteins was converted to a binary table using a perl script and imported into R. Ade4 package was used to perform the principal-component analysis (PCA) [> data_pca <- dudi.pca(table.binary.tsv, scale = F, scannf = FALSE, nf = 5)]. The plot was created using the two first principal components, and the strains were colored based on their virulence levels (red, high virulence; green, low virulence; gray, unknown virulence phenotype) {> plot(data_pca$li[,1:2], col=color_t_condition, pch =19)}.

### Data availability.

All genomic sequences are publicly available through NCBI (BioProject accession PRJNA634019).
