# Supplementary figures and images for: Combining Whole-Genome Sequencing and Multimodel Phenotyping To Identify Genetic Predictors of Salmonella Virulence
Source: mSphere. 2020 Jun 10;5(3):e00293-20. doi: 10.1128/mSphere.00293-20 (PMC7289705; doi:10.1128/mSphere.00293-20)

Figure S1

A

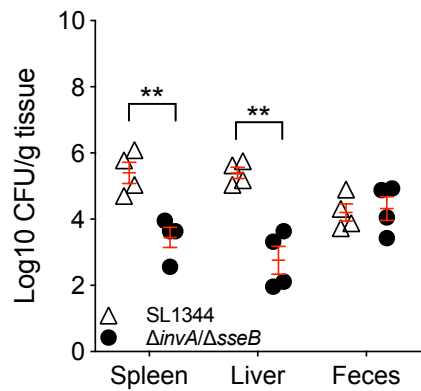

B

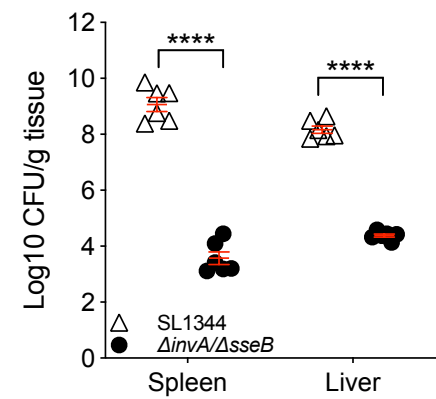

C

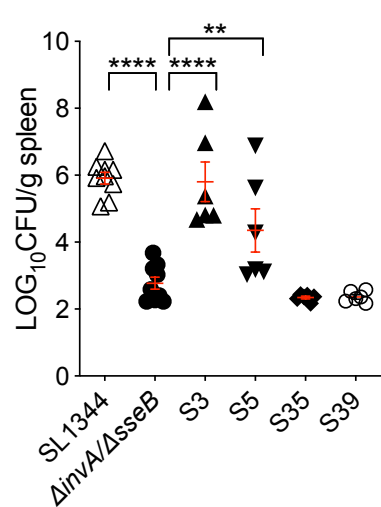

D

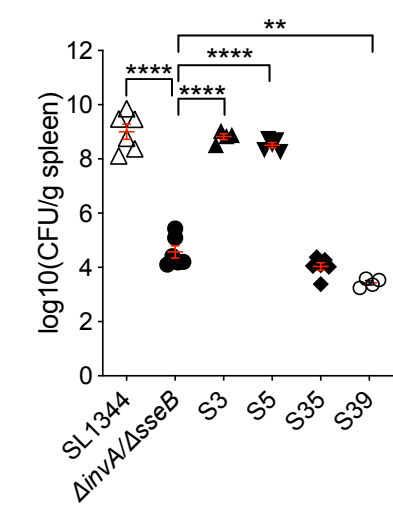

Supplement: FIG S1 [file mSphere.00293-20-sf001.pdf]

Figure S2

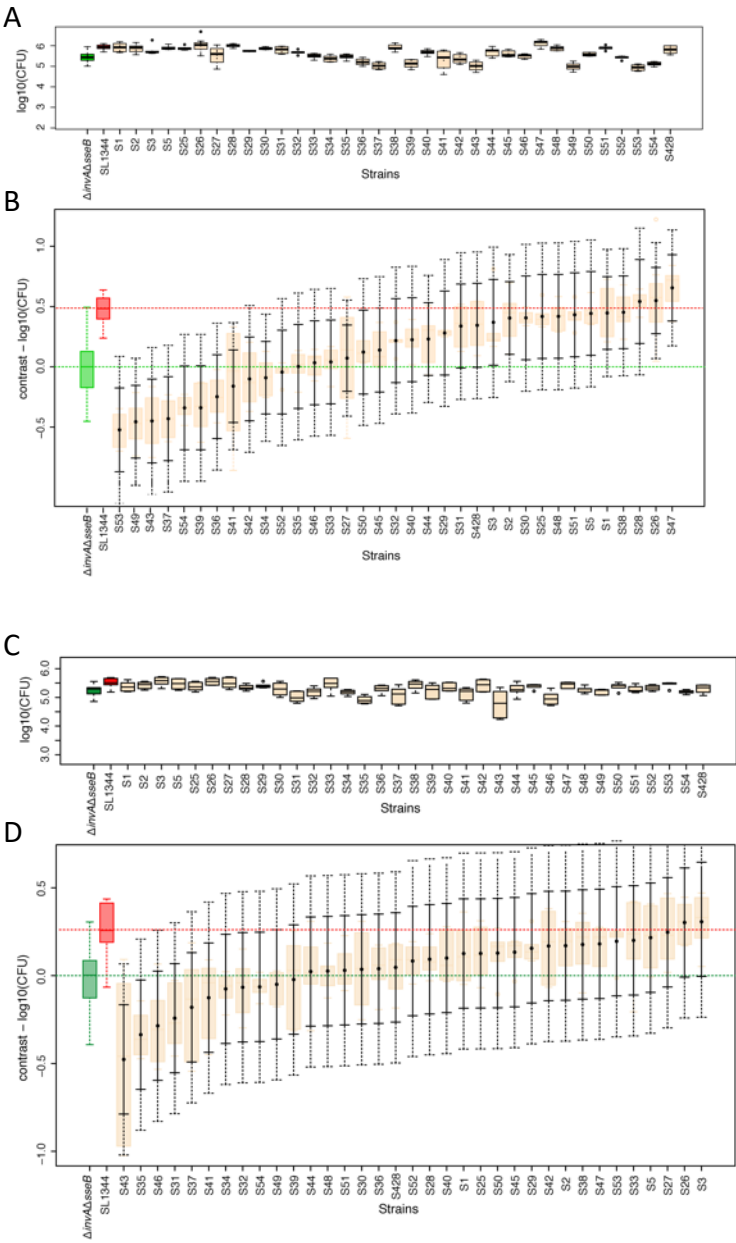

Supplement: FIG S2 [file mSphere.00293-20-sf002.pdf]

Figure S3

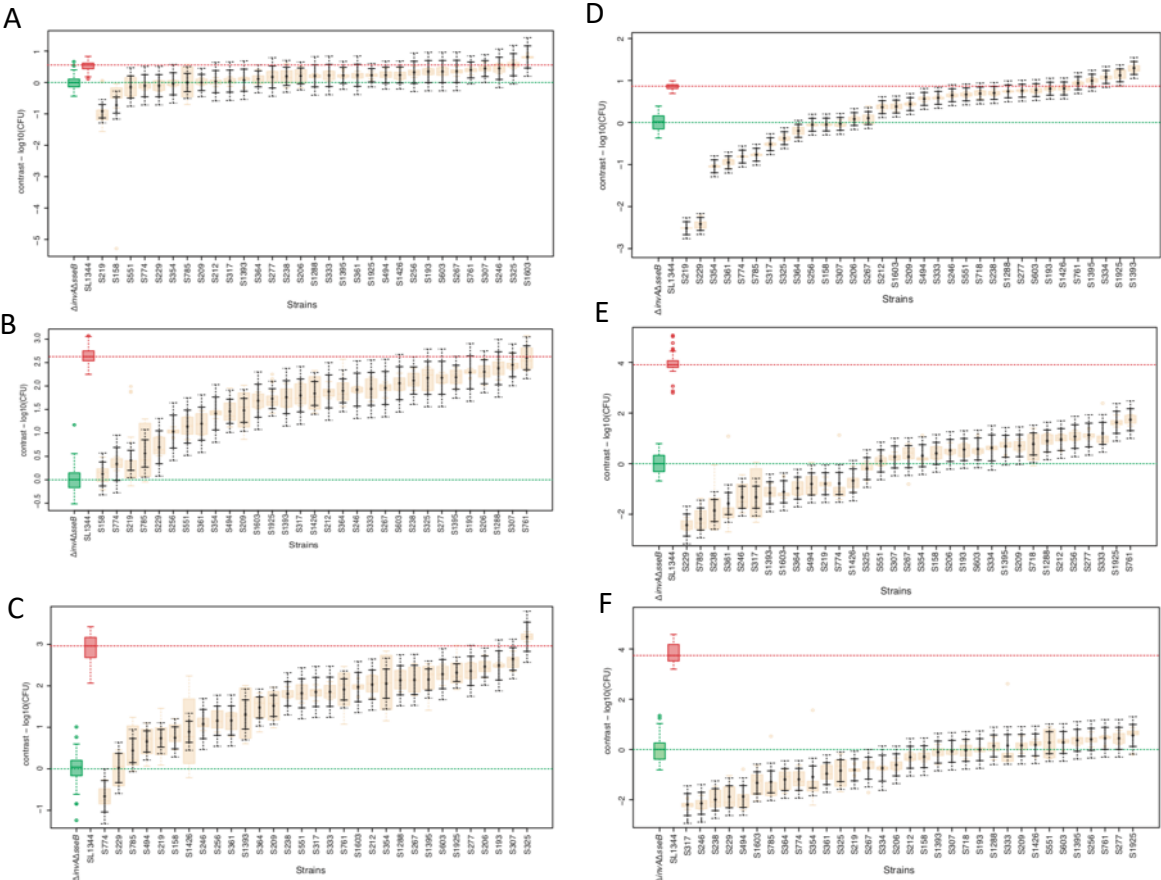

Supplement: FIG S3 [file mSphere.00293-20-sf003.pdf]

Figure S4

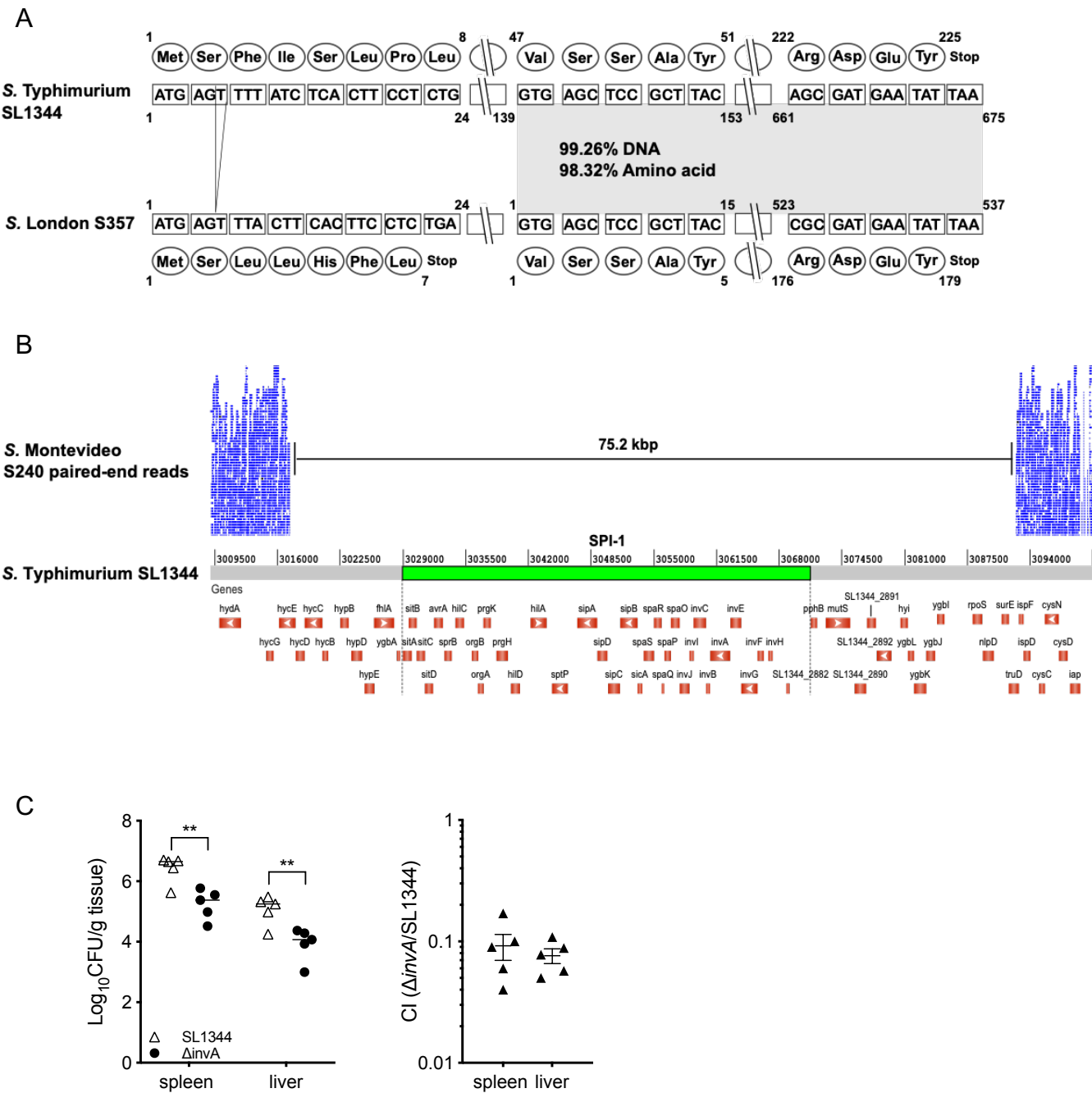

Supplement: FIG S4 [file mSphere.00293-20-sf004.pdf]
